# Supplementary material for: Prostate-specific antigen dynamics predict individual responses to intermittent androgen deprivation
Source: Nat Commun. 2020 Apr 9;11:1750. doi: 10.1038/s41467-020-15424-4 (PMC7145869; doi:10.1038/s41467-020-15424-4)
Supplement: Supplementary file 1 — Supplementary Information [file 41467_2020_15424_MOESM1_ESM.pdf]

Supplementary Information for

Prostate-Specific Antigen Dynamics Predict Individual Responses to Intermittent Androgen Deprivation

Supplementary Figure 1

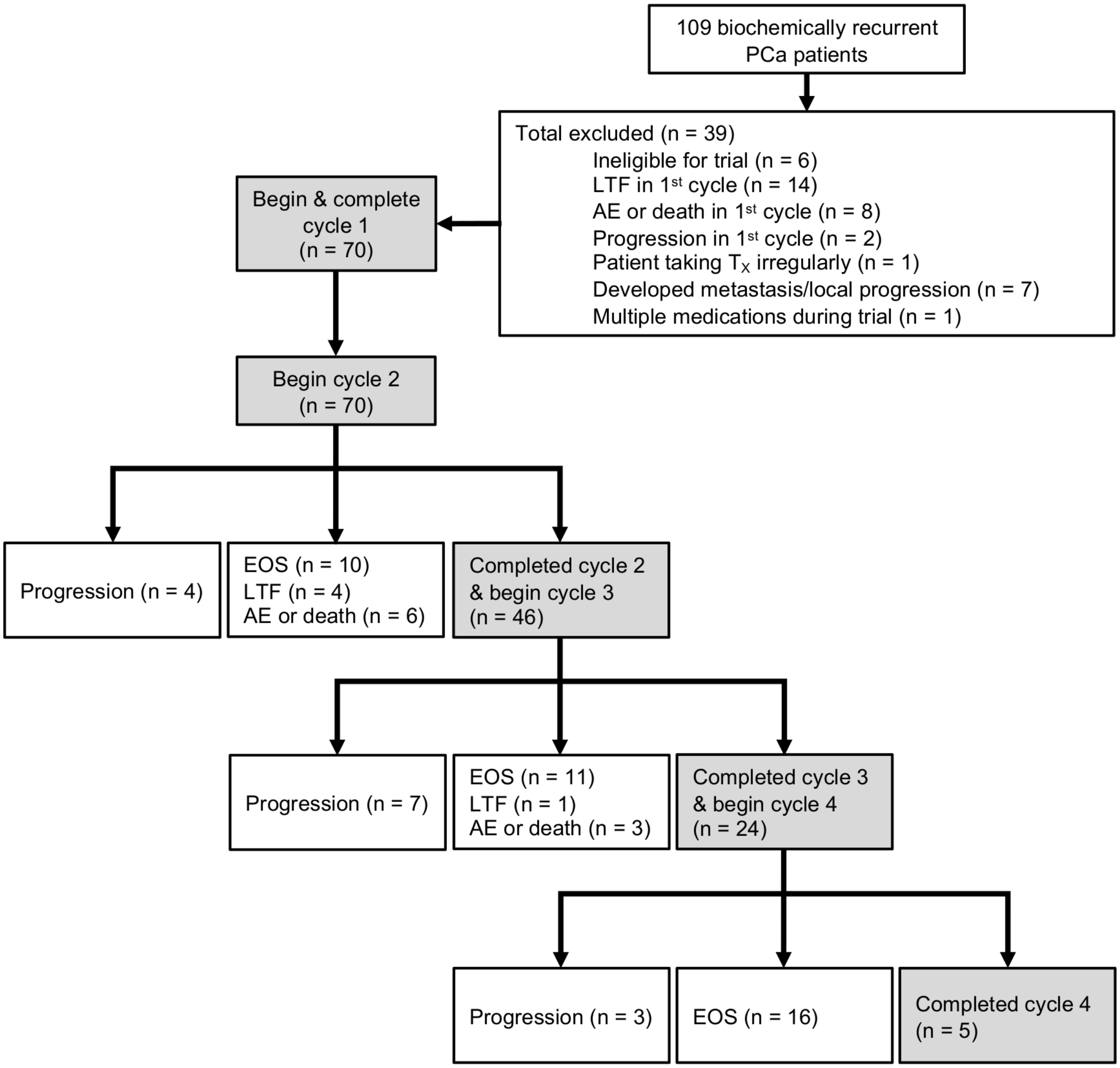

Data stratification for biochemically recurrent PCa patients enrolled in trial by Bruchovsky et al. Of the 109 patients enrolled in the trial, 70 were included in the analysis. EOS, LTF, and AE denote end of study, lost to follow up, and adverse event, respectively.

Supplementary Figure 2

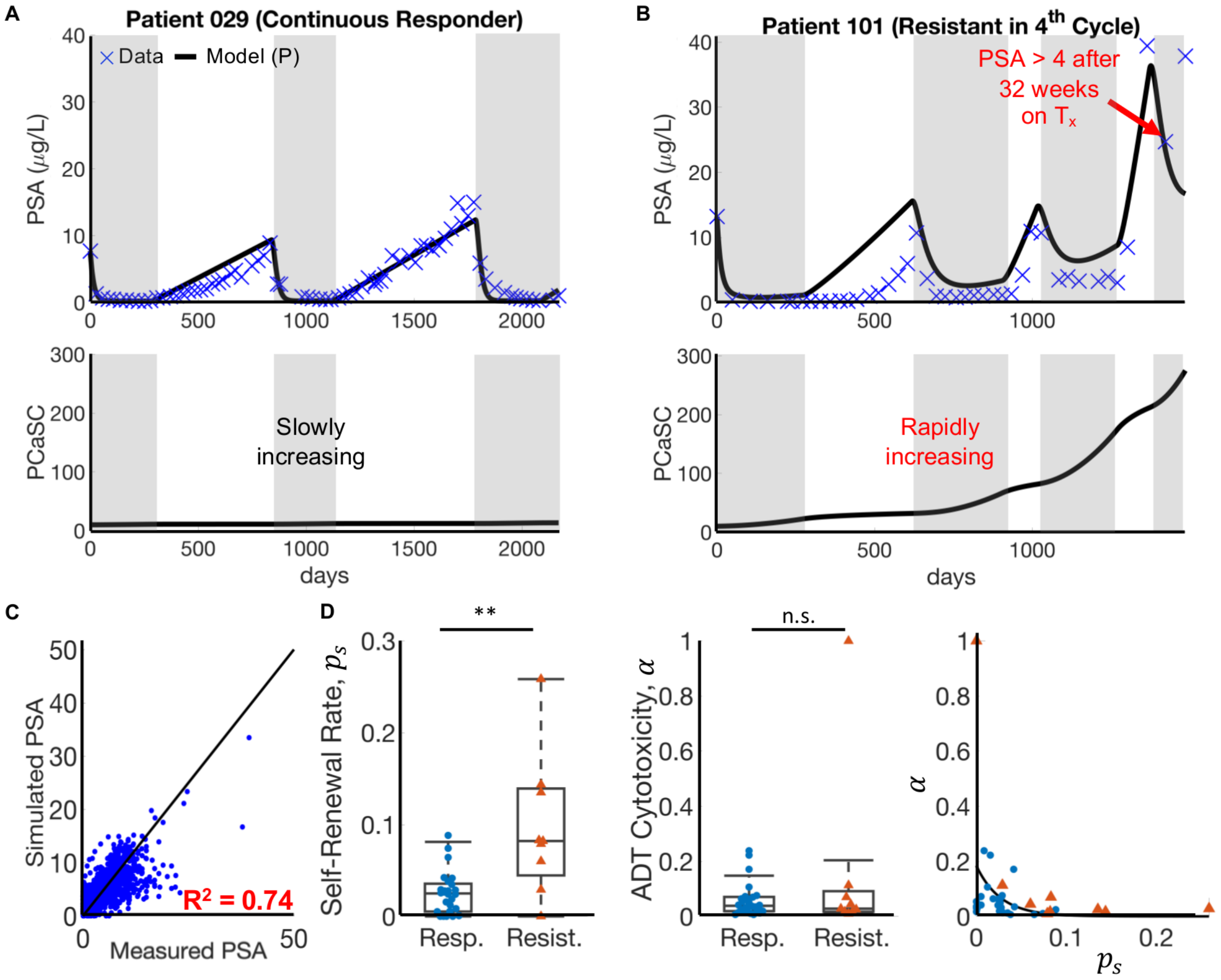

Parameter distributions and model fits for training patients. (**A** and **B**) Model fits to PSA data and corresponding PCaSC dynamics for (A) a continuous responder and (B) a patient who developed resistance during his fourth cycle of treatment. PCaSC population is rapidly increasing in the resistant patient and slowly in the responsive patient due to a significantly higher self-renewal rate (p_s = 0.0052 and 0.1349 for patients 029 and 101, respectively). **(C)** Simulated vs. measured PSA. Linear regression obtains an R^2 of 0.74. **(D)** Parameter distributions, comparing between responsive (n = 26, blue dots) and resistance (n = 9, red triangles) patients with φ and ρ uniform between all training patients. Stem cell self-renewal p_s and ADT cytotoxicity α exhibit exponential relationship. The two-sample t-test was used to calculate the statistical significance of the difference between the two groups. Boxplots show median including the 25th and 75th percentiles. The two-sample t-test was used to calculate the statistical significance of the difference between the two groups (p = 0.009 [left pane, significance level denoted by double star], p = 0.422 [right pane, not significant (n.s.)]). In (D), blue dots and red triangles denote responsive and resistant patients, respectively.

Supplementary Figure 3

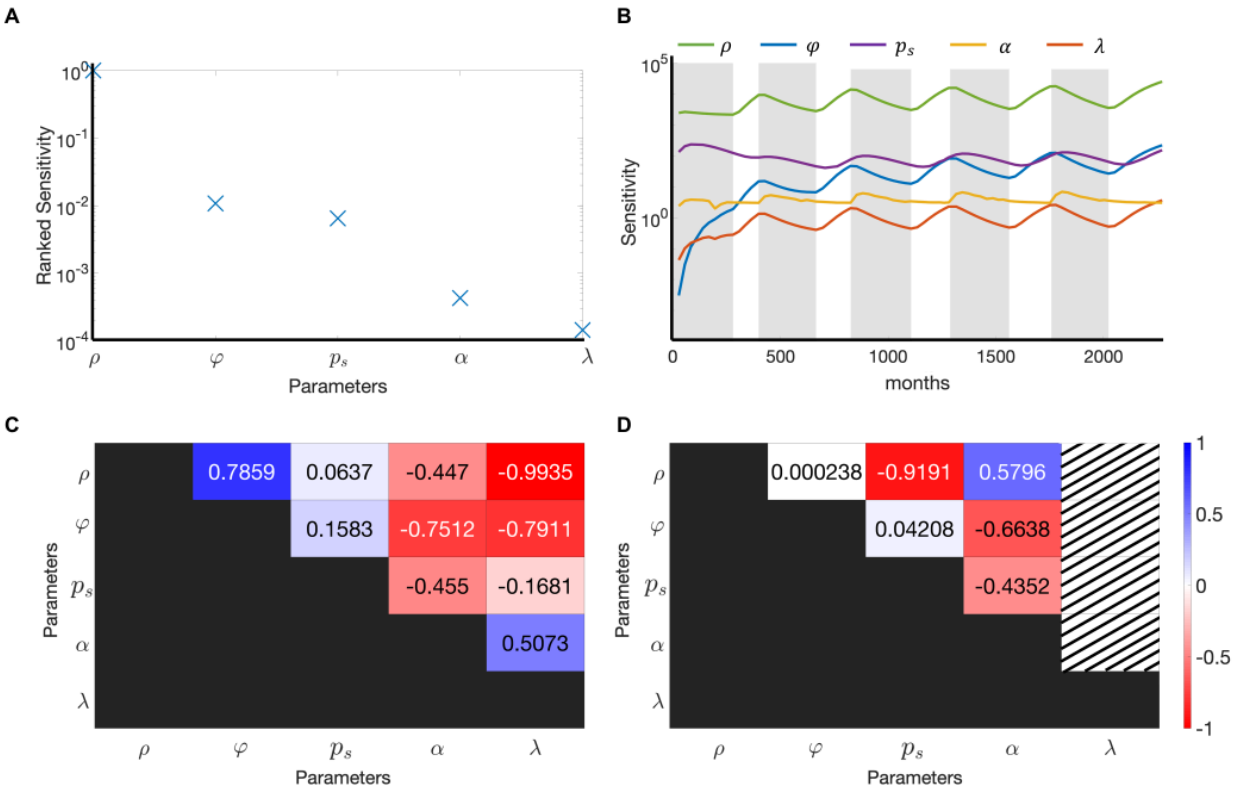

Model sensitivity analysis. (A) Ranked sensitivities of the five parameters of the model. λ has the lowest sensitivity in comparison of the other parameters. **(B)** Sensitivity of each parameter with respect to time for one patient. Sensitivities for each parameter varies throughout each treatment cycle. **(C)** Correlation analysis for model parameters. Analysis shows that λ is negatively correlated with ρ and φ (correlation coefficient c = -0.9935 and -0.7911, respectively). **(D)** As the least sensitive parameter, λ was removed and the correlations were recomputed. Though the analysis shows that p_s and ρ are negatively correlated, the correlation coefficient, c = -0.9191, was less than the set correlation cutoff $\xi = 0.95$. Thus, the sensitive, uncorrelated parameters are ρ, φ, p_s, and α.

Supplementary Figure 4

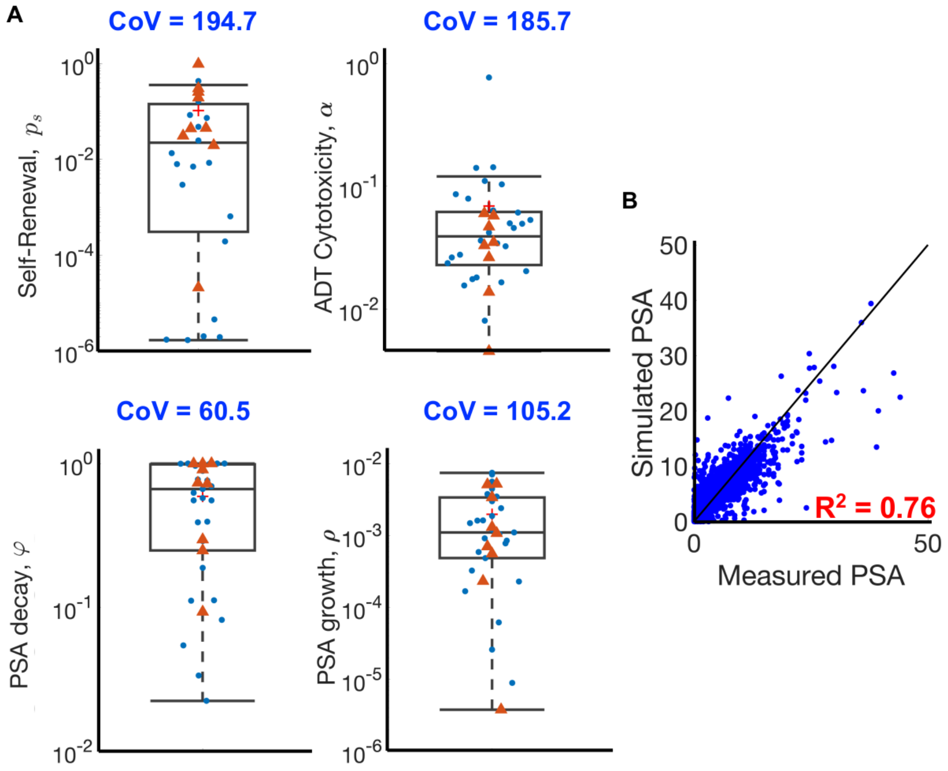

Optimization results when all sensitive and uncorrelated parameters are optimized. (A) Parameter distributions for p_s, α, φ, and ρ with the corresponding coefficients of variation. Responsive patients (n = 26) and resistance patients (n = 9) are shown by blue dots and red triangles, respectively. Boxplots show median including the 25th and 75th percentiles. The two-sample t-test was used to calculate the statistical significance of the difference between the groups. **(B)** Simulated vs. measured PSA for all patients when four parameters are optimized.

Supplementary Figure 5

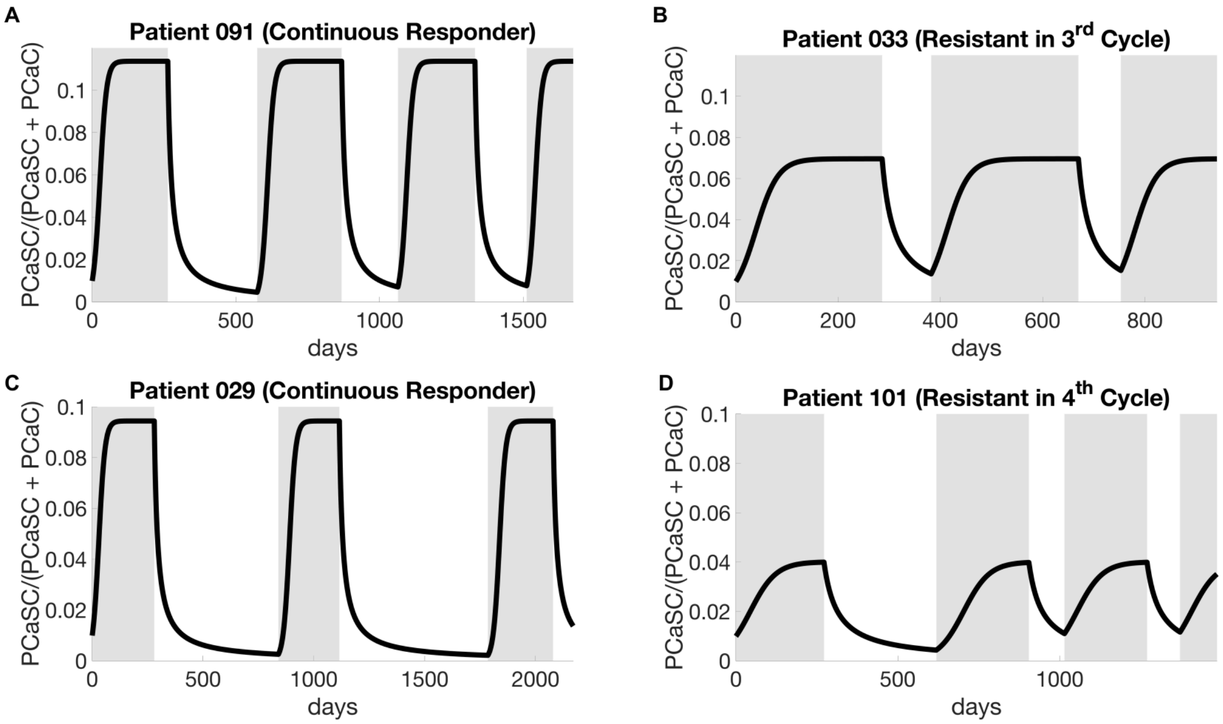

Stem cell proportion dynamics. (**A-D**) Proportion of stem cells for two responsive patients (A and C) and two resistant patients (B and D). Gray shaded area denotes when treatment is on. The PCaSC proportion increases to a steady state during treatment and decays when treatment is turned off. As a patient develops resistance, the proportion of PCaSCs decreases to a higher steady state during each off cycle, whereas the responsive patients maintain the values that the PCaSC proportion decreases to between cycles. The change in the proportion of stem cells $\bar{s} = \frac{S}{S+D}$ can be expressed as $\frac{d\bar{s}}{dt} = \alpha T_x \bar{s}(1-\bar{s}) - (1-p_s)\lambda\bar{s}^2$.

Supplementary Table 1

α vs. p$_s$ relationship comparison. Investigation of possible functional relations between α and p_s. Comparison of mean squared error (MSE) over all data for each function.

**Equation**	**MSE** $\frac{1}{n}\sum\left(y_m^i - y_d^i\right)^2$
$\alpha = A + Bp_s$	0.03
$\alpha = Ae^{-Bp_s}$	0.02
$\alpha = A - B\ln(p_s)$	0.02
$\alpha = A + \frac{B}{p_s}$	6E19

Supplementary Table 2

Optimal parameter, threshold, and overall accuracy 95% confidence intervals for five additional randomizations. Confidence intervals were obtained by re-randomizing the patients into training and testing cohorts, optimizing all patients in the training cohort to find uniform values for the PSA production and decay rates and then evaluating the predictive ability of the model.

**Optimal Parameters**		
φ	ρ	
[0.0336, 0.0634]	[5.382E-05, 1.288E-04]	

**Thresholds**			**Overall Accuracy**
κ_2	κ_3	κ_4	[75.34, 88.66]
[0.283, 0.430]	[0.231, 0.325]	[0.094, 0.319]	

Supplementary Table 3
Optimal parameter, threshold, and overall accuracy 95% confidence intervals for leave-one-out study. Bootstrapping leave-one-out study was completed for all 70 patients. Optimization and forecasting analysis were completed and the predictive ability of the model was evaluated.

<table>
<tr><th colspan="6">Optimal Parameters</th></tr>
<tr><th colspan="3">φ</th><th colspan="3">ρ</th></tr>
<tr><td colspan="3">[0.013, 0.019]</td><td colspan="3">[2.877E-05, 3.761E-05]</td></tr>
<tr><th colspan="6">Thresholds</th></tr>
<tr><th colspan="2">κ_2</th><th colspan="2">κ_3</th><th colspan="2">κ_4</th></tr>
<tr><td colspan="2">[0.299, 0.343]</td><td colspan="2">[0.348, 0.391]</td><td colspan="2">[0.221, 0.356]</td></tr>
</table>
