# Supplementary material for: Prostate-specific antigen dynamics predict individual responses to intermittent androgen deprivation
Source: Nat Commun. 2020 Apr 9;11:1750. doi: 10.1038/s41467-020-15424-4 (PMC7145869; doi:10.1038/s41467-020-15424-4)
Supplement: Supplementary file 3 — Reporting summary [file 41467_2020_15424_MOESM3_ESM.pdf]

nature research

Corresponding author(s): Heiko Enderling
Robert A. Gatenby

Last updated by author(s): Feb 25, 2020

Reporting Summary

Nature Research wishes to improve the reproducibility of the work that we publish. This form provides structure for consistency and transparency in reporting. For further information on Nature Research policies, see Authors & Referees and the Editorial Policy Checklist .

Statistics

For all statistical analyses, confirm that the following items are present in the figure legend, table legend, main text, or Methods section.

n/a	Confirmed	
☐	☒	The exact sample size (*n*) for each experimental group/condition, given as a discrete number and unit of measurement
☒	☐	A statement on whether measurements were taken from distinct samples or whether the same sample was measured repeatedly
☐	☒	The statistical test(s) used AND whether they are one- or two-sided<br>*Only common tests should be described solely by name; describe more complex techniques in the Methods section.*
☒	☐	A description of all covariates tested
☒	☐	A description of any assumptions or corrections, such as tests of normality and adjustment for multiple comparisons
☐	☒	A full description of the statistical parameters including central tendency (e.g. means) or other basic estimates (e.g. regression coefficient) AND variation (e.g. standard deviation) or associated estimates of uncertainty (e.g. confidence intervals)
☒	☐	For null hypothesis testing, the test statistic (e.g. *F*, *t*, *r*) with confidence intervals, effect sizes, degrees of freedom and *P* value noted<br>*Give P values as exact values whenever suitable.*
☒	☐	For Bayesian analysis, information on the choice of priors and Markov chain Monte Carlo settings
☒	☐	For hierarchical and complex designs, identification of the appropriate level for tests and full reporting of outcomes
☒	☐	Estimates of effect sizes (e.g. Cohen's *d*, Pearson's *r*), indicating how they were calculated

Our web collection on statistics for biologists contains articles on many of the points above.

Software and code

Policy information about availability of computer code

Data collection	No software was used to collect the data.
Data analysis	All analysis was completed in MATLAB v2019a. Code supporting the findings of this study are available at https://github.com/reneebrady/IADT_PCaSC.

For manuscripts utilizing custom algorithms or software that are central to the research but not yet described in published literature, software must be made available to editors/reviewers. We strongly encourage code deposition in a community repository (e.g. GitHub). See the Nature Research guidelines for submitting code & software for further information.

Data

Policy information about availability of data

All manuscripts must include a data availability statement. This statement should provide the following information, where applicable:

- Accession codes, unique identifiers, or web links for publicly available datasets
- A list of figures that have associated raw data
- A description of any restrictions on data availability

The clinical data used to conduct this study are available in a public repository at http://www.nicholasbruchovsky.com/clinicalResearch.html.

Field-specific reporting

Please select the one below that is the best fit for your research. If you are not sure, read the appropriate sections before making your selection.

☒ Life sciences ☐ Behavioural & social sciences ☐ Ecological, evolutionary & environmental sciences

Life sciences study design

All studies must disclose on these points even when the disclosure is negative.

Sample size	The data was equally divided, using stratified random sampling, into a training set (n = 35) and testing set (n = 35).
Data exclusions	Of the 109 patients, 39 were excluded due to ineligibility for the trial (n = 6), lost to follow-up, adverse event, death, or progression during the first cycle (n = 24), irregular or multiple medications during trial (n = 2), or metastasis development (n = 7).
Replication	The study was reproduced by switching the testing and training sets and rerunning the analysis. A bootstrapping leave-one-out analysis was done to better account for under-represented resistant patients in the training cohort. This process was repeated for all patients individually (70 times)
Randomization	Stratified random sampling was used to randomize the data into training and testing sets. Leave-one-out analysis was done to better account for the under-represented resistant population of patients.
Blinding	The investigators were blinded to group allocation during data analysis.

Reporting for specific materials, systems and methods

We require information from authors about some types of materials, experimental systems and methods used in many studies. Here, indicate whether each material, system or method listed is relevant to your study. If you are not sure if a list item applies to your research, read the appropriate section before selecting a response.

Materials & experimental systems

n/a	Involved in the study	
☒	☐	Antibodies
☒	☐	Eukaryotic cell lines
☒	☐	Palaeontology
☒	☐	Animals and other organisms
☐	☒	Human research participants
☐	☒	Clinical data

Methods

n/a	Involved in the study	
☒	☐	ChIP-seq
☒	☐	Flow cytometry
☒	☐	MRI-based neuroimaging

Human research participants

Policy information about studies involving human research participants

Population characteristics	Initial study conducted by Bruchovsky et al. DOI: 10.1002/cncr.21989
Recruitment	Initial study conducted by Bruchovsky et al. DOI: 10.1002/cncr.21989
Ethics oversight	Initial study conducted by Bruchovsky et al. DOI: 10.1002/cncr.21989

Note that full information on the approval of the study protocol must also be provided in the manuscript.

Clinical data

Policy information about clinical studies

All manuscripts should comply with the ICMJEguidelines for publication of clinical research and a completedCONSORT checklist must be included with all submissions.

Clinical trial registration	Initial study conducted by Bruchovsky et al. DOI: 10.1002/cncr.21989
Study protocol	Initial study conducted by Bruchovsky et al. DOI: 10.1002/cncr.21989
Data collection	Initial study conducted by Bruchovsky et al. DOI: 10.1002/cncr.21989
Outcomes	Initial study conducted by Bruchovsky et al. DOI: 10.1002/cncr.21989
